# Optimization and partial purification of beta-galactosidase production by *Aspergillus niger* isolated from Brazilian soils using soybean residue

**DOI:** 10.1186/s13568-019-0805-6

**Published:** 2019-06-10

**Authors:** Raquel Dall’Agnol Martarello, Luana Cunha, Samuel Leite Cardoso, Marcela Medeiros de Freitas, Damaris Silveira, Yris Maria Fonseca-Bazzo, Mauricio Homem-de-Mello, Edivaldo Ximenes Ferreira Filho, Pérola Oliveira Magalhães

**Affiliations:** 1 Laboratory of Natural Products, Health Sciences School, Department of Pharmaceutical Sciences, University of Brasília, Brasília, DF CEP 7910-900 Brazil; 2 Laboratory of Enzymology, Department of Cellular Biology, University of Brasilia, Brasília, DF CEP 70910-900 Brazil

**Keywords:** Fungi, β-Galactosidase, Purification, Optimization, Agroindustrial residues

## Abstract

β-Galactosidases are widely used for industrial applications. These enzymes could be used in reactions of lactose hydrolysis and transgalactosylation. The objective of this study was the production, purification, and characterization of an extracellular β-galactosidase from a filamentous fungus, *Aspergillus niger.* The enzyme production was optimized by a factorial design. Maximal β-galactosidase activity (24.64 U/mL) was found in the system containing 2% of a soybean residue (w/v) at initial pH 7.0, 28 °C, 120 rpm in 7 days. ANOVA of the optimization study indicated that the response data on temperature and pH were significant (p < 0.05). The regression equation indicated that the R^2^ is 0.973. Ultrafiltration at a 100 and 30 kDa cutoff followed by gel filtration and anion exchange chromatography were carried out to purify the fungal β-galactosidase. SDS-PAGE revealed a protein with molecular weight of approximately 76 kDa. The partially purified enzyme showed an optimum temperature of 50 °C and optimum pH of 5.0, being stable under these conditions for 15 h. The enzyme was exposed to conditions approaching gastric pH and in pepsin’s presence, 80% of activity was preserved after 2 h. These results reveal a *A. niger* β-galactosidase obtained from residue with favorable characteristics for food industries.

## Introduction

Fermentation of agroindustrial residues received a great deal of attention in recent years. Many byproducts and raw materials from the food industry and agriculture, e.g., soybean residues, sugarcane bagasse, cotton stalk, corn cob, and mango peel have been used to produce biotechnological products owing to their high availability. They are also an alternative source of nutrients with low commercial cost (Moreira et al. 2012).

The agroindustrial residues are mainly composed of lignocellulosic material. Considering that 90% of agroindustrial residues are discarded into the environment, the use of these residues as raw materials should reduce environmental pollution and may also increase the economic value of the residues (Moreira et al. 2012).

Brazil is the second biggest producer of soybeans (*Glycine max*) worldwide. In the 2014–2015 harvest, soybean planting area reached 30.1 million hectares, producing a crop of 95 million tons of soybeans (Embrapa 2016). One application for soybean byproducts is fermentation by microorganisms including bacteria and fungi that are able to degrade the lignocellulosic material of the agricultural residues. These residues could be utilized by filamentous fungi as a carbon source for the production of enzymes, in particular hydrolytic ones (Moreira et al. 2012). *Aspergillus* fungi have been chosen for large-scale processes because they can produce large quantities and varieties of enzymes in a low-cost medium (Bergquist et al. 2002). Moreira et al. (2012) studied the degradation of lignocellulosic residues for production of enzymes of industrial significance such as xylanases, mannanases, pectinases, β-glucosidases, avicelases, phosphatases, and carboxymethyl cellulases by different species of fungi isolated from soil, including *Aspergillusterreus*, *Aspergillusoryzae*, and *Aspergillusniger* (Moreira et al. 2012).

A large number of microorganisms have been assessed as potential sources of β-d-galactosidase (β-d-galactoside galactohydrolase, EC 3.2.1.23, most commonly known as lactase) to hydrolyze lactose into glucose and galactose for lactose-free milk production and products intended for lactose-intolerant consumers (Isobe et al. 2013a). Traditionally, the β-galactosidases most widely used in industry were obtained from *Aspergillus* spp. and *Kluyveromyces* spp. (Panesar et al. 2006), because these could be readily obtained with acceptable productivities and yields from cultivations of these microorganisms. Additionally, products obtained from these organisms are generally recognized as safe (GRAS status) for human consumption, which is critical for food related applications (Panesar et al. 2006). In *Aspergillus niger* the β-galactosidase enzymes are secreted to the extracellular medium, increasing the interest in finding new culture source for the production of this enzyme (Panesar et al. 2006). Besides, lactose is a hygroscopic sugar that has low solubility; it could induce crystallization and may cause technological problems for certain products in the dairy industry. The solubility and sweetness can be increased by the lactose hydrolysis. Many problems in refrigerated foods such as crystallization in dairy foods, precipitate formation in frozen foods, and development of a gritty texture may be reduced with lactose hydrolysis (Klein et al. 2010; Panesar et al. 2006).

β-Galactosidases also participate in the synthesis of galactooligosaccharides (GOSs) and can be applied to functional foods such a slow-calorie foods or as an additive in fermented dairy products, breads, and drinks.

Moreover, in the pharmaceutical industry, β-galactosidase is produced as a food supplement for lactose-intolerant people. Many symptoms of lactose intolerance are minimized by the use of exogenous β–galactosidase before ingestion of milk or dairy products (O’Connell and Walsh 2008; Oliveira et al. 2011).

Optimization of the fermentation process can reduce the production costs for β-galactosidase and is important for enabling an industrial application and to obtain “green processes.” For this reason, the selection of a low-cost culture is fundamental (Liu et al. 2007). Optimization of media components by the traditional “one-variable-at-a-time” strategy is time-consuming and expensive when a large number of variables are considered. This method is incapable of detecting the true optimum, especially because of the interactions between the factors (Hajji et al. 2008). Recently, a number of statistical designs were successfully employed for optimization of enzyme production by microorganisms. Response surface methodology (RSM) is one of the most popular optimization procedures, developed mainly from the factorial central composite design (CCD). Furthermore, CCD minimizes the risk of losing a nonlinear relation between intervals and enables researchers to estimate a reasonable model and check the fit of the model. These statistical techniques have been successfully applied in many studies for optimization of a culture medium (Patel et al. 2005). These techniques are available using analysis of variance (ANOVA), which aims to reveal how much of total variance exists between and within the groups evaluated. If most of the variance is between the groups, then there is probably a significant effect. However, if most of the variance is within the values of the same group, there is probably no significant effect. The RSM could be used after ANOVA to correlate the effect of independent variables (Rodrigues and Iemma 2005). The objective of this study was to apply experimental models to increase galactosidase production followed by purification with chromatographic and non-chromatographic methods and then enzymatic characterization.

The industrial search for alternative technologies that have high productivity, consume less resources, and have a lesser environmental impact is intensifying worldwide (Jegannathan and Nielsen 2013). Thus, the search for new fungal β-galactosidase sources produced from a soybean residue with industrial applicability is becoming interesting from biotechnological, economic, and environmental points of view.

## Materials and methods

### Chemicals

The chemicals ONPG (*o*-nitrophenol-β-d-galactopyranoside), *o*-nitrophenol, and other reagents were purchased from Sigma-Aldrich Chemical Co. (St. Louis, MO, USA). The media components used in this study were the following: peptone from Merck (Darmstadt, Germany), and potato dextrose agar (PDA) and yeast extract from HiMedia Laboratories Pvt. Ltd. (Mumbai, India).

### Residue pretreatment

The soybean residue was autoclaved at 121 °C for 2 h and thoroughly washed with tap water. After that, it was dried at 65 °C for 48 h and then ground into a relatively homogeneous blend. A fine powder was obtained and utilized as the carbon source. To obtain a liquid extract of soybeans (soymilk) and okara, 250 g of whole soybeans were washed and soaked in tap water for 12 h; this step is called maceration. At the next step, the soybeans were washed again and cooked for 10 min in 1 L of boiling water. After that, the soybeans were washed with running water, passed through a mesh and ground for 3 min in 1 L of boiling water. Next, the ground beans were filtered through a cotton cloth strainer. The filtered liquid was the soy extract (soymilk), and the solid residue is called okara. The residue was then dried at 65 °C to suitable humidity of approximately 3–5%, macerated, and stored at room temperature. The liquid extract was stored at − 20 °C until use.

### The microorganism and culture conditions

*Aspergillus niger* strain was isolated from soil samples of the Brazilian biome Cerrado and deposited under strain code DCFS11 in the fungal culture collection at the Enzymology Laboratory, University of Brasilia, Brazil (genetic heritage number 010237/2015-1). The strain was also deposited in the bank of microorganisms for control of plant pathogens and weeds of the Brazilian Agricultural Research Corporation (EMBRAPA). The collection is registered at the World Data Centre for Microorganisms (WDCM), under the code MCPPW 1128. It was preserved in 0.9% NaCl (w/v), 50% glycerol (v/v), and 0.01% Tween 80 (v/v) solution at − 80 °C and propagated on potato dextrose agar plates at 28 °C. The isolate was initially subjected to morphological identification according to Silva et al. (2018).  The internal transcribed spacer region (ITS) and genes for β-tubulin (BT) and calmodulin (CMD) were used as molecular markers to confirm fungal identity (Silva et al. 2018).

### Enzyme production

Initial screening for the most significant carbon source was performed via the one-variable-at-a-time approach. The carbon sources included soybean residue, okara, and soymilk (2%, w/v) and were supplemented with 0.4% of peptone, 0.4% of yeast extract, 0.2% of KH_2_PO_4_, 0.8% of NaH_2_PO_4_, and 0.25% MgSO_4_ at initial pH 7.0. Cultures were inoculated with 10^7^ spores/mL in 250 mL Erlenmeyer flasks with a working volume of 50 mL of culture media and were incubated on a rotatory shaker (120 rpm) at 28 °C for 7 days. The culture medium and mycelia were harvested by vacuum filtration through Whatman No. 1 filter paper on a Büchner funnel. The obtained supernatant was utilized for crude extract preparation.

### Enzymatic assay

β-Galactosidase activity was determined by the method proposed by Nagy et al. (2001). The crude extract (0.05 mL) was mixed with 0.05 mL of 3 mM *o*-nitrophenol-β-d-galactopyranoside (ONPG) dissolved in 0.1 M sodium phosphate buffer (pH 7.0), and incubated at 30 °C for 30 min. The reaction was stopped by adding 2.0 mL of a 0.1 M sodium carbonate solution (Na_2_CO_3_). One unit (U) of β-galactosidase activity was defined as the amount of the enzyme catalyzing the release of 1 µmol of *o*-nitrophenol per min according to measurement of absorbance at 410 nm. Protein concentration was measured by the Bradford method (Bradford 1976), with bovine serum albumin as a standard.

### The experimental design

To characterize how the significant factors, affect the responses, we attempted to improve composition of the medium by comparing different levels of several factors that were found to have more influence on the β-galactosidase production. According to the results of the one-variable-at-a-time experiments, the effects of three factors, agitation speed (*X*_1_), temperature (*X*_2_), and pH (*X*_3_), were found to be the major variables affecting enzyme production in the medium containing the soybean residue. For this purpose, RSM involving a central composite design (CCD) was employed for optimization of the enzyme production. In this regard, a set of 11 experiments, including 2^3^ factorial experiments with three center points, were carried out. Each variable was studied at two different levels (− 1, + 1) and at a center point (0), which is the midpoint of each factor range (Table 1). The response values (*Y*) in each trial were averages of triplicates.

**Table 1:** Variables values of a 2^3^ factorial design with centered face and three repetitions at the central point used to evaluate the influence of (X_1_) speed agitation, (X_2_) temperature and (X_3_) pH on β-galactosidase production

Variables	Symbol code	Levels		
		− 1	0	1
Speed agitation (rpm)	*X*_1_	100	120	140
Temperature (^o^C)	*X*_2_	22	28	34
pH	*X*_3_	5	7	9

The experimental results of RSM were fitted via the response surface regression procedure, by means of the following second-order polynomial equation:

$$Y = \alpha_{0} + \mathop \sum \limits_{i} \alpha_{i} X_{i} + \mathop \sum \limits_{ij} \alpha_{ij} X_{i} X_{j}$$

where *Y* is the predicted response; *α*_*0*_ is an intercept term; *α*_*i*_ is a linear coefficient; *α*_*ii*_ is a quadratic coefficient; and *α*_*ij*_ are interaction coefficients. *X*_*i*_ and *X*_*j*_ are the levels of the independent variables.

A statistical software package, Statistica 7.0, was used for the regression analysis of the experimental data and to plot the response surface graphs. Only the factors with significance higher than or equal to 95% (*p* < 0.05) were considered. The quality of fit of the second-order polynomial model equation was expressed via the coefficient of determination (*R*^*2*^) and adjusted *R*^*2*^. The fitted polynomial equation was then expressed in the form of three-dimensional surface plots, to illustrate the correlation between the responses and the experimental levels of each variable utilized in this study. The point optimization method was employed to optimize the level of each variable for a maximum response. The combination of different optimized variables that yielded a maximum response was determined in an attempt to validate the model.

### Enzyme purification

The *A. niger* culture was filtered, and the supernatant was ultrafiltered through membranes with a 100 and 30 kDa cutoff. Subsequent assays were conducted on a purification system (AKTA PURE; GE Healthcare). The obtained enzyme solution was eluted on a Sephacryl S-200 column (16 × 60 cm) equilibrated with 0.1 M sodium phosphate buffer, pH 7.0. The sample eluted at a fixed flow rate of 0.5 mL/min was collected in 2 mL fractions. Fractions with β-galactosidase activity were pooled and lyophilized.

The lyophilized sample from the gel filtration column was dissolved in 0.1 M sodium phosphate buffer, pH 7.0. The sample was passed through a DEAE Fast Flow anion exchange column (DEAE FF-GE Healthcare; 1 mL) equilibrated with the same buffer at a flow rate of 0.25 mL/min. A linear gradient of sodium chloride (0.0 to 1 M) was applied.

### Enzyme characterization

#### Electrophoresis (SDS-PAGE) and a zymogram

The protein profile of the denatured samples was analyzed in a 10% polyacrylamide gel stained with a Coomassie blue solution. For the zymogram, the gel obtained by the electrophoretic run of a nondenatured sample was immediately incubated in a buffer consisting of a solution of 5-bromo-4-chloro-indolyl-β-d-galactopyranoside (X-Gal) at 0.02% (w/v) (O’Connell and Walsh 2008).

#### Effects of pH and temperature on enzymatic activity

The pH effect on β-galactosidase activity was estimated in the presence of buffer solutions: potassium chloride–hydrochloric acid (pH 1.0 to 1.5), 50 mM sodium acetate (pH 2.0 to 6.0), and 50 mM sodium phosphate (pH 7.0 to 9.0) and the activity was measured as described above (Enzymatic assay). The optimal temperature of the enzyme was evaluated at 4 °C, 10 °C, 15 °C, 20 °C, 30 °C, 40 °C, 50 °C, 60 °C, 70 °C, and 80 °C. Enzymatic activity was assessed as described above (Enzymatic assay).

#### Thermostability

The samples were incubated at 50 °C for 20 h. The assay was performed at pH 3.0. The residual enzymatic activity was determined every 60 min, as in the enzymatic assay described above (Enzymatic assay).

#### Stability under gastric conditions

Simulation of gastric digestion was conducted by a modified method of O’Connell and Walsh (2008). We incubated 5 mL of the enzyme with 5 mL of simulated gastric fluid (SGF), pH 2.0 for 2 h at 37 °C and 150 rpm. The SGF was prepared as per Brazilian Pharmacopoeia and consisted of a solution containing 0.2% of NaCl, 0.32% of purified bovine pepsin (Sigma-Aldrich, USA), and 7.0% HCl. A control composed of the buffer and enzyme and another control consisting of the buffer and SGF were set up too.

#### Determination of the kinetic parameters of β-galactosidase

The Michaelis–Menten constant (K_m_) and tests for maximum velocity (V_max_) determination were performed at substrate concentrations (ONPG) ranging from 1 to 50 mM, and the substrate lactose concentrations ranging from 1 to 300 mM. Activity assays were performed as described above by replacing the ONPG with the substrates at the concentrations mentioned above. The results on the kinetic parameters were obtained in the Enzfitter software (Leatherbarrow 1999).

## Results

### The effect of the soybean residue on β-galactosidase production

**Fig. 1:**
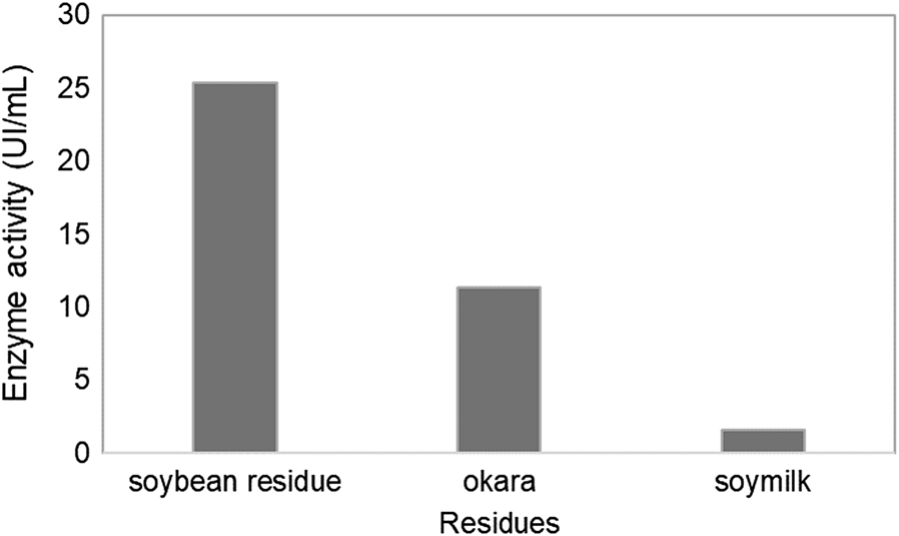
β-galactosidase activity in different soybean products, after 7 days of submerse fermentation at 120 rpm and 28 °C

The *A. niger* strain was grown in a liquid medium supplemented with the soybean residue, okara, or soymilk for 7 days. The highest activity was observed in the medium with the soybean residue when compared with okara and soymilk, as shown in Fig. 1. Considering its low β-galactosidase activity, the growth in soymilk medium was rejected from this study.

Among the residues tested as carbon sources, the soybean residue was the most suitable substrate for β-galactosidase production. Thus, this substrate was selected for further optimization steps.

### Evaluation of β-galactosidase production

In the present study, CCD was implemented along with the corresponding results of RSM experiments to determine the effects of three independent variables (Table 2). The results showed a maximal β-galactosidase activity (24.64 U/mL) at the central point of the 2^3^ full factorial design. The best conditions involving the soybean residue as a substrate were found at 120 rpm, 30 °C, and pH 7.0.

**Table 2:** Results of β-galactosidase activity (U/mL), according to the 2^3^ factorial design with centered face and three repetitions at the central point central

Run orders	Coded levels			β-Galactosidaseactivity (U/mL)^a^
	*X*_1_	*X*_2_	*X*_3_	
1	− 1	− 1	− 1	19.64
2	1	− 1	− 1	19.02
3	− 1	1	− 1	7.07
4	1	1	− 1	8.83
5	− 1	− 1	1	10.06
6	1	− 1	1	0.03
7	− 1	1	1	0.00
8	1	1	1	1.12
9	0	0	0	23.4
10	0	0	0	24.64
11	0	0	0	22.60

Data: (*X*_*1*_) speed agitation, (*X*_*2*_) temperature and (*X*_*3*_) pH on β-galactosidase production

^a^One unit (U) of β-galactosidase activity was defined as the amount of the enzyme catalyzing the release of 1 µmol of o-nitrophenol per min at 410 nm (Nagy et al. 2001)

The ANOVA indicated that the response data for temperature ($X_{2}$) and pH ($X_{3}$) were significant (*p* < 0.05), but the interactions of the variables were not significant. Significance of coefficients has been reported to be directly proportional to the *t* value and inversely to the *p* value. Thus, the high significance of these variables indicates that this parameter can act as a limiting factor, and even small variations in its value will alter β-galactosidase activity to a considerable extent. Therefore, the regression equation coefficients were calculated in terms of actual factors. The data were fitted to a second-order polynomial equation, and the response (*Y*), i.e., β-galactosidase production by *A. niger*, could be expressed as follows: where $X_{1} , X_{2} , {\text{and}}\,X_{3}$ are agitation speed, temperature, and pH, respectively.

The regression equation obtained by ANOVA (Table 3) indicated that the multiple correlation coefficient *R*^2^ is 0.973 (value greater than 0.75 indicates suitability of the model), i.e., the model can explain 97.3% of the variation response. The *F* value of the model was 18.21, implying that the model is significant. The *P* values for the model (0.018) and for a lack of fit (0.0945) suggested that the data were a good fit for the model. The ANOVA provided a satisfactory adjustment of the model to the experimental data. The results indicated that the model could be used to predict the β-galactosidase activity.

**Table 3:** Analysis of variance (ANOVA) for β-galactosidase activity

Source	*F* value	*p*-value > *F*
Model	18.21	0.018
Speed agitation	1.92	0.259
Temperature	32.17	0.010
pH	60.04	0.004
*X*_1_ *X*_2_	5.83	0.094
*X*_1_ *X*_3_	3.22	0.170
*X*_2_ *X*_3_	6.07	0.090
Lack of fit	9.110	0.094

The model including as independent variables (X1) agitation, (X2) temperature and (X3) pH

The speed of agitation has no statistically significant influence on β-galactosidase production. On the other hand, pH and temperature, among the analyzed factors, interfere with enzymatic activity. The temperature and pH have a negative influence on β-galactosidase activity. The predicted negative sign (–) of variables ${\text{X}}_{1}$ and ${\text{X}}_{3}$ indicated that an increase in temperature or pH tended to reduce β-galactosidase activity and the interactions among variables ${\text{X}}_{1}$, ${\text{X}}_{3}$, ${\text{X}}_{1} {\text{X}}_{2} ,$ and ${\text{X}}_{2} {\text{X}}_{3}$ were not significant.

**Fig. 2:**
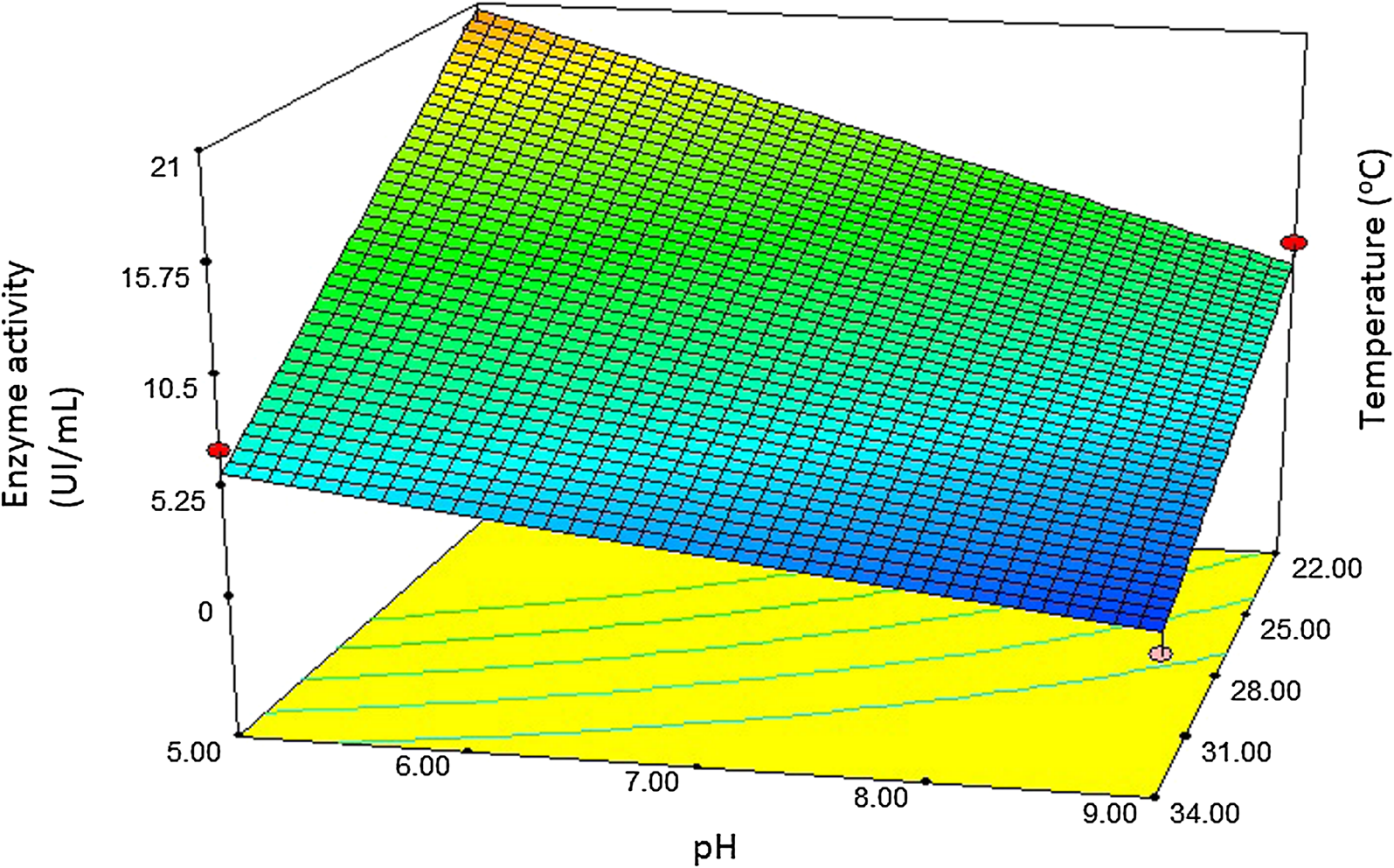
Response surface curve of β-galactosidase activity affected by temperature and pH using the 2-factor central composite design

To determine the optimal levels of each variable for maximal β-galactosidase production by *A. niger*, three-dimensional response surface graphs were constructed by plotting the response (enzyme production) on the *z*-axis against two independent variables, while maintaining other variables at their optimal levels. The Fig. 2 shows the effects of temperature and pH on β-galactosidase production in a medium containing the soybean residue.

### Construction of the growth curve for *A. niger* reflecting β-galactosidase activity

**Fig. 3:**
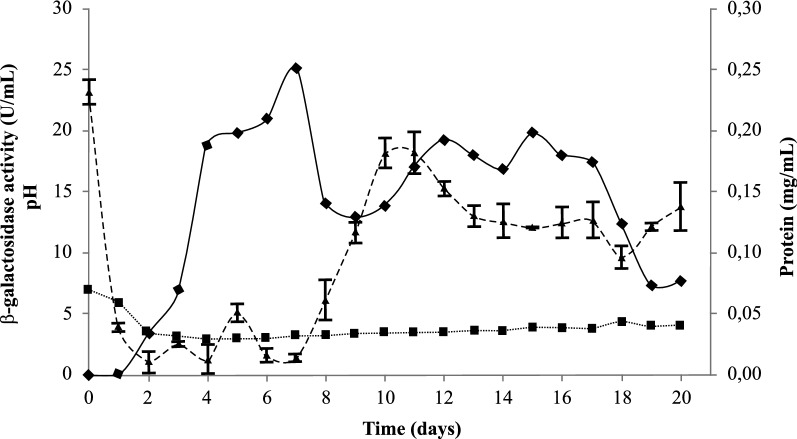
Time courses of β-galactosidase production by *Aspergillus niger* under optimized conditions (120 rpm, 28 °C, initial medium pH 7), using medium supplemented with 2% of soybean residue. Medium pH (∙∙∙∙), protein (mg/mL) (---) and β-galactosidase activity (—)

The growth curve of the β-galactosidase production by *A. niger* under the best conditions obtained by RSM was constructed after monitoring the data for 20 days. As presented in Fig. 3, after 4 days of fermentation, the production of β-galactosidase increased dramatically. This production reached a maximum (25.11 U/mL) after 7 days of fermentation in the medium supplemented with the soybean residue.

The amount of protein changed during the culture period, suggesting that this result may include other enzymes besides β-galactosidase, which are simultaneously produced and participate in the substrate degradation process. The induction profile followed pH variation, with a maximum value of 5.92 on the 1st day of culture and minimum value of 2.94 on the 4th day.

### β-Galactosidase purification

The crude extract was concentrated approximately 2-fold at 4 °C by ultrafiltration on an Amicon membrane System (Amicon Inc., Beverly, MA, USA) with a 100 and 30 kDa cutoff (Table 4).

**Table 4:** Summary of purification steps of β-galactosidase from *A. niger*

Purification steps	Protein (mg/mL)	Activity (UI/mL)	Specific activity (UI/mg)	Yield (%)	Purification (fold)
Crude	0.150	26.000	173.333	100.000	1.000
< 100	0.144	24.000	166.667	92.308	0.962
> 30	0.065	23.440	360.615	90.154	2.080
S-200	0.0075	6.570	876.000	25.269	5.054
DEAE	0.003	4.506	1502.000	17.331	8.665

The concentrated fraction (> 30) was eluted from an S-200 chromatographic column and yielded only one β-galactosidase activity peak 1S-200. Peak 1S-200 was eluted from an anionic ion exchange column (HiTrap DEAE-FF 1 mL) resulting in two activity peaks. The first one (β-gal peak) showed the highest activity (1502 UI/mg protein) and was selected for the following tests. The final purification factor with two chromatographic steps was 8.665 and the yield was 17.33% (Table 5).

**Table 5:** Substrate specificity of β-galactosidase partially purified from *Aspergillus niger*

Substrate^a^	Activity (UI/mL)^b^
ONPG	22.125 ± 0.018
Lactose	5.622 ± 0.002
CM-cellulose	0.253 ± 0.004
ρNPG	0.043 ± 0.005

^a^ONPG: *o*-nitrophenol-β-d-galactopyranoside; ρNPG: ρ-nitrophenol-β-d-galactopyranoside; CM-cellulose: carboxymethyl celulose; Lactose: β-d-galactopyranosyl-(1→4)-d-glucose

^b^One unit (U) of β-galactosidase activity was defined as the amount of the enzyme catalyzing the release of 1 µmol of *o*-nitrophenol per min at 410 nm (Nagy et al. 2001)

**Fig. 4:**
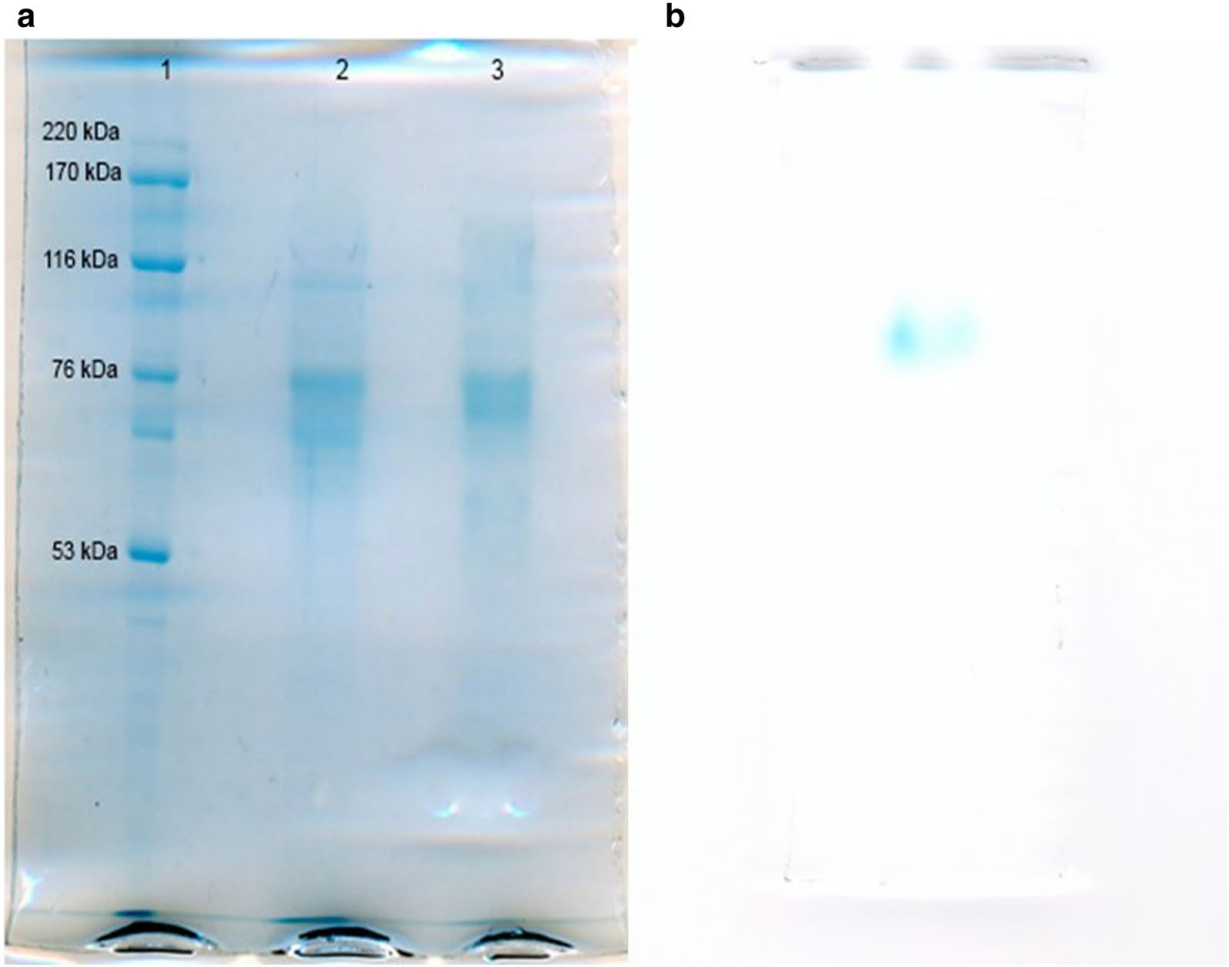
**a** SDS-PAGE of partially purified β-galactosidase. Lane 1: Molecular mass markers, mass indicated alongside. Lane 2: S-200 peak. Lane3: DEAE peak. **b** β-galactosidase activity zymogram from DEAE peak

The protein profiles of each step of *A. niger* β-galactosidase purification were followed by electrophoresis. The Fig. 4 shows an electrophoretic run of S-200 (2) and DEAE (3).

In fraction (2), only two bands were observed with estimated molar masses of 76.58 and 70.25 kDa. Fraction (2) refers to a single activity peak present in the chromatogram (β-gal peak; Fig. 4), suggesting that in the partially purified fraction, there are two proteins with very close molecular weight and similar physical and chemical characteristics. Therefore, it was concluded that the column was not able to separate these two proteins. On the other hand, when we analyzed the zymogram (Fig. 4), there was a single blue band characteristic of β-galactosidase activity in X-Gal (Kishore and Kayastha 2012).

### Enzymatic characterization

#### Effects of temperature and pH on enzymatic activity

**Fig. 5:**
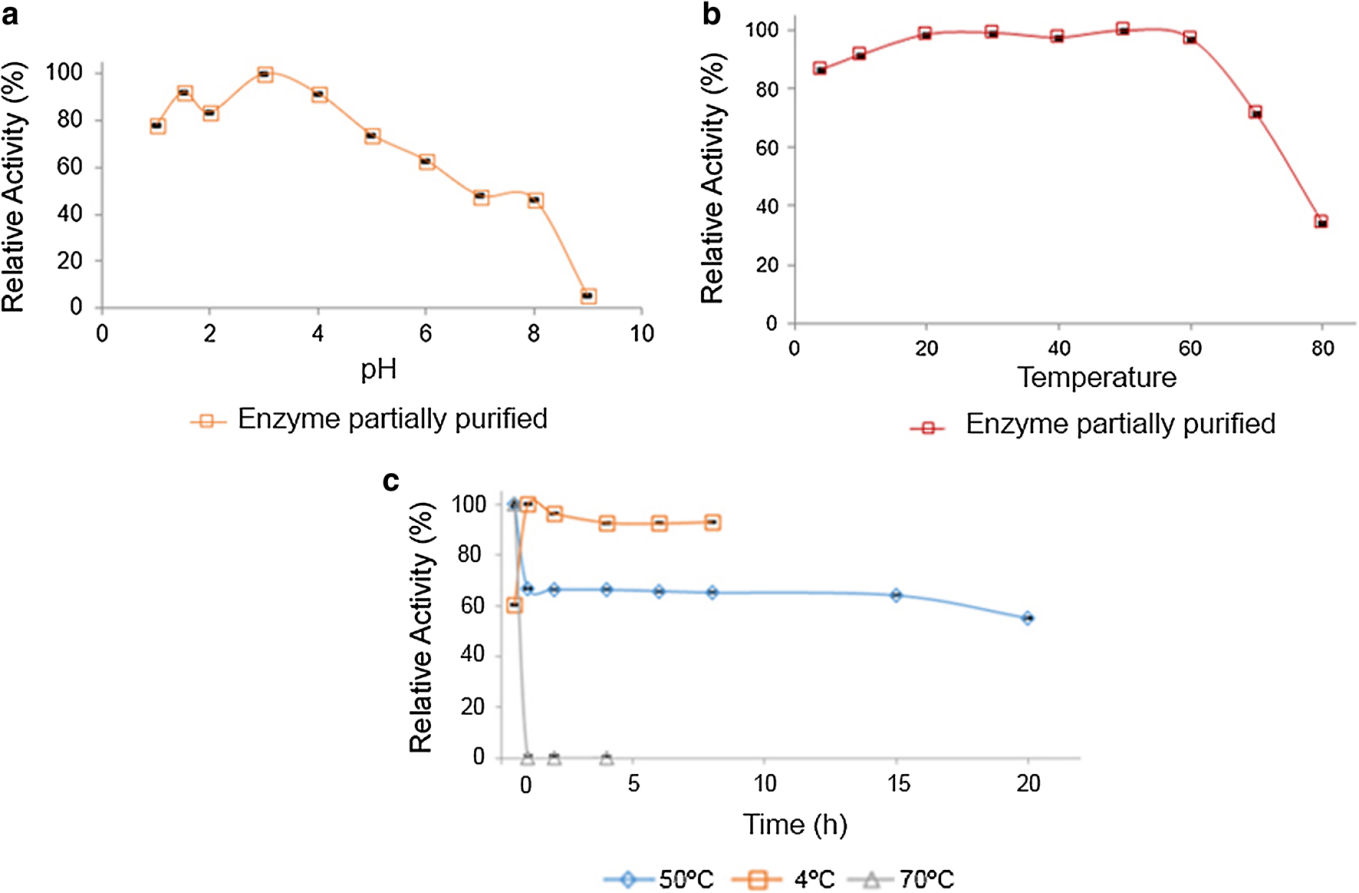
**a** Activity versus pH profiles of the partially purified β-galactosidase. Enzyme activity is plotted as a % value relative to the activity displayed at the enzyme’s optimum pH. Error bars indicate the standard deviation of the measured data values from the mean, n = 3. **b** Activity versus temperature profiles of the partially purified β-galactosidase. Enzyme activity is plotted as a % value relative to the activity displayed at the enzyme’s optimum temperature. Error bars indicate the standard deviation of the measured data values from the mean, n = 3. **c** Temperature stability of the β-galactosidase partially purified from *A. niger* at 4 °C, 50 °C and 70 °C

β-Gal showed a higher activity between pH 2.0 and 5.0, being more active at pH 3.0 (Fig. 5a). This enzyme needs to be active in acid pH values during lactose digestion, since gastric pH is known to be 1.5–2.5. Acidic β-galactosidases are important for biotechnology with applications in the whey processing and yogurts. In addition, there is a high probability of resistance to gastric conditions enabling its use as food supplements for the lactose-intolerant population (O’Connell and Walsh 2008).

The influence of temperature on the β-gal activity is presented in Fig. 5b. The activity remained constant between 20 °C and 60 °C, indicating the industrial applicability of this enzyme (O’Connell and Walsh 2008).

#### Thermal stability

Our aim is application of this enzyme to food products such as milk and derivatives. It is interesting from the industrial point of view that the enzyme is stable both at low temperatures (preventing proliferation of microorganisms and preserving nutrients in milk) and at high temperatures (pasteurization). Thus, the stability of the enzyme persisted at temperatures of 4 °C, 50 °C, and 70 °C as depicted in Fig. 5c.

The partially purified enzyme was subjected to a thermostability assay in which residual β-galactosidase activity was measured after an incubation period. After 1 h incubation at 70 °C, there was no more enzymatic activity, but when incubated at 50 °C for 1 h, the enzymatic activity decreased by 33.3% and persisted for approximately 15 h. When incubated at 4 °C, the enzymatic activity persisted, indicating good stability at this temperature, which shows the possibility of the use in lactose hydrolysis in low temperatures.

#### Substrate specificity and kinetic constants

Specificity of the enzyme was evaluated by a hydrolysis assay involving several substrates (Table 4). The enzyme was active in the hydrolysis of ONPG and lactose. Toward the other substrates, the enzyme showed little or no activity.

The β-galactosidase showed activity mostly toward ONPG and lactose, indicating that the enzyme holds great promise for application in the dairy industry, which requires enzymes without action on other substrates.

In addition to specificity, the kinetic parameters (K_m_ and V_max_) of β-galactosidase at 50 °C for the substrates ONPG and lactose were analyzed according to the Michaelis–Menten model. K_m_ and V_max_ for ONPG counts were 1.84 mM and 256.65 IU/mL, respectively, whereas for lactose, K_m_ was 40 mM and V_max_ 2.86 IU/mL.

#### Stability under simulated gastric conditions

**Fig. 6:**
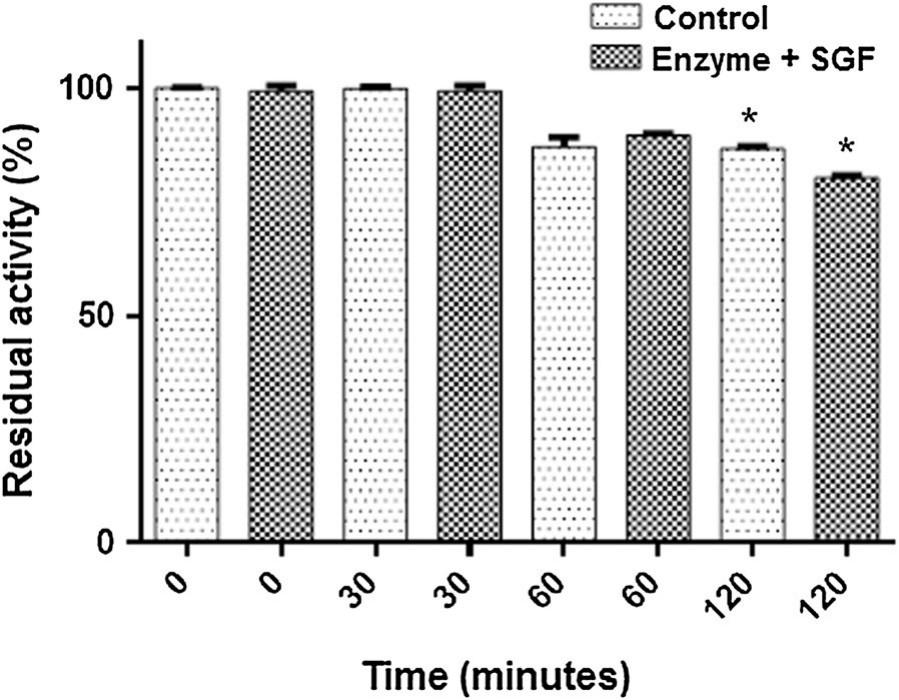
The effect of in vitro simulated fasted gastric (SGF) conditions on partially purified β-galactosidase. Control: enzyme incubated with buffer pH 2.0. Enzyme + SGF (NaCl 2%, bovine pepsin 0.32% and HCl 7%). *Indicates statistical difference in the ANOVA Tukey test with significance level (p < 0.05) between control and enzyme + SGF

Because of possible industrial applicability of β-galactosidase as a digestive supplement for lactose-intolerant individuals, evaluation of the enzyme activity was performed under simulated gastric conditions. The enzymatic activity is presented as a percentage (%) relative to the activity of the control, where 100% is equated to 0.5 IU/mL. Figure 6 shows that β-galactosidase had a residual activity of 80% when subjected to SGF for 2 h.

## Discussion

### β-Galactosidase production

Soybean agroindustrial residues are important for the biotechnology industry as an abundant low-cost source for enzymatic production. The use of soybean residues reduces environmental pollution. For this reason, this study evaluates the production of β-galactosidases by *A. niger* from soybean residues as a substrate.

Several β-galactosidases secreted by the fungus *Aspergillus niger* have been purified and characterized. The occurrence of this enzyme in multiple molecular forms has been suggested by previous work (O’Connell and Walsh 2008; Hu et al. 2010). Studies have shown that other microorganisms can express enzymes when cultivated with organic residues such as soybean residues (de Siqueira et al. 2010).

O’Connell and Walsh (2008) reported that 0.91 U/mL β-galactosidase activity is produced by *Aspergillus carbonarius* cultivated in wheat bran (O’Connell and Walsh 2008). In other study, *A. niger* grown with the soybean residue yielded β-galactosidase activity 60% greater than did *A. niger* grown with the okara residue (O’Toole 1999). A decrease in enzymatic production in the okara residue medium can be attributed to the presence of phytic acid. The chelating ability of phytic acid present in okara prevents the use of micronutrients present in the culture medium. This chelating agent can scavenger minerals such as zinc, calcium, and iron, among others, by binding these metals, thereby making them insoluble and difficult to absorb by the fungus (O’Toole 1999).

RSM can be implemented to examine the relation between experimental factors and the observed results; furthermore, it is the most widely accepted statistical technique for bioprocess optimization. At the end of the screening experiments for different carbon sources, three factors (agitation speed, temperature, and medium pH) were believed to play a significant role in β-galactosidase production and were selected for RSM.

According to Rahman et al. (2005), temperature also affects the synthesis of extracellular enzymes by causing changes in physical properties of the cell membrane (Rahman et al. 2005). Sudharhsan et al. (2007) also demonstrated the importance of pH and temperature for the synthesis and secretion of microbial amylases (Sudharhsan et al. 2007).

In this study, RSM was employed to optimize culture conditions for enzyme production using a soybean residue as a carbon source. The best conditions for β-galactosidase production by the filamentous fungus *A. niger* at the evaluated parameters were initial medium pH 7.0, agitation speed of 120 rpm, temperature of 28 °C, and fermentation duration of 7 days. Among the various nutrient sources tested, the minimum medium with 2% (w/v) of the soybean residue was sufficient for maximal enzyme production. The medium supplemented with the soybean residue is economically viable, with a good potential for the biotechnological industry. Khayati et al. (2014) has studied beta-galactosidase production from indigenous and inexpensive wastes under SSF conditions using statistical experimental design Methods. Design of experimental methodology using Taguchi orthogonal array was applied to evaluate the influence of five factors (peanut pod concentration, C/N ratio, incubation time, type of solid substrate and lactose concentration) on the β-galactosidase production by *Bacillus licheniformis* under solid-state fermentation. The results showed that peanut pod concentration, incubation time and lactose (as inducer) were found to be the most effective factor for promoting enzyme production, followed by the C/N ratio.

The result obtained regarding the enzymatic activity of β-galactosidase in this work was higher than or similar the other studies found in the literature involving different culture media and different microorganisms. Meanwhile, Braga et al. (2012) obtained a β-galactosidase activity of 10.4 U/mL from *Kluyveromyces marxianus* grown in a medium containing a rice effluent (Braga et al. 2012_ENREF_38). Nagy et al. (2001) obtained β-galactosidase activity of 14.31 U/mL from *Penicillium chrysogenum* in a fermentation medium containing lactose. Braga et al. (2012) achieved an activity of 10.4 U/mL expressed by *K. marxianus* in an effluent from rice cultivation. Chuming Nie et al. (2013) were able to produce higher β-galactosidase activity, 24.5 and 31 U/mL, by means of recombinant lactobacilli systems in *Pichia pastoris* (Nie et al. 2013).

### β-Galactosidase purification

Literature data reveal some strategies for purifying β-galactosidases from filamentous fungi. Some methodologies involve several chromatography steps resulting in multiple yield and purification factor levels. Nagy et al. (2001) purified a fungus β-galactosidase from a *Penicillium chrysogenum* strain on an ion exchange column and affinity column. Their study achieved a purification index of 66 and a yield of 8% (Nagy et al. 2001). O’Connell and Walsh (2008) purified two isoforms of β-galactosidase from *A. carbonarius* reaching a purification factor of 6 and 3 and a yield of 5% and 1% by gel chromatographic techniques with ion exchange and hydrophobic columns (O’Connell and Walsh 2008). Isobe et al. (2013a; b) purified two β-galactosidase subunits from *Teratosphaeria acidotherma* reaching a purification factor and yield of 375 and 2.9%, respectively. In that study, the authors used ion exchange chromatography (DEAE), gel filtration, and hydrophobic and affinity columns (Isobe et al. 2013b).

Nonetheless, a smaller number of purification steps are important for reducing the cost and losses of a target compound. In many cases, the costs of these operations can reach 80% of the total cost of production.

Dimeric β-galactosidases with similar molecular weight that were observed in this study are consistent with the data reported in the literature for different fungi and bacteria β-galactosidases. Isobe et al. (2013a; b) found a fungal β-galactosidase of 140 kDa with two subunits of 86 and 50 kDa (Isobe et al. 2013b). Nagy *et al*. (2001) reported that the β-galactosidase from *Penicillium chrysogenum* is a multimeric enzyme of ~270 kDa composed of 66 kDa monomers (Nagy et al. 2001). Kong et al. (2014) cloned the β-galactosidase of *Thermotoganaphthophila* and expressed it in *E. coli*. The purified recombinant enzyme showed a molecular weight of 70 kDa (Kong et al. 2014). The fungal β-galactosidase in this work was obtained with few purification steps from a low-cost residue, thus being valuable for the industry from the economic point of view.

### β-Galactosidase characterization

Our results are in agreement with the literature, where Lima et al. (1982) purified an *A. niger* β-galactosidases with an optimum temperature of 65 °C and optimum pH of 4.5 (Lima et al. 1982). Generally, β-galactosidases produced by fungi present optimal pH in the acidic range and have relatively high optimal temperature (O’Connell and Walsh 2008). Gekas and Lopez-Leiva (1985) reported that an *A. niger* β-galactosidase was characterized and found to have an optimum pH between 3.0 and 4.0 and optimum temperature between 55 and 60 °C. *A. oryzae* β-galactosidase shows an optimum pH of 5.0 and optimum temperature between 50 °C and 55 °C (Gekas and Lopez-Leiva 1985). The isoforms of β-galactosidase from *A. carbonarius* during submerged fermentation were described by O’Connell and Walsh (2008), showing optimal pH values of 3.0 and 5.0 and optimum temperatures of 55 °C and 65 °C (O’Connell and Walsh 2008). It was observed that in an *A. niger* strain studied by Niu et al (2017) the enzyme produced has an optimum pH between 4 and 5 and an optimum temperature of 50 °C that supports the results found for β-galactosidase studied in this work (Niu et al. 2017). These results for temperature and pH are similar to other reported in the literature for β-galactosidases from *Aspergillus* spp. β-galactosidase from *A. lacticoffeatus* presented an optimal temperature in the range 50–60, and remained quite active for temperatures between 35 °Cand 65 °C. The optimal pH for this β-galactosidase was found in the range 3.5–4.5, and the enzymatic activity decreased significantly for higher pH values (Cardoso et al. 2017). β-Galactosidase from *Aspergillus nidulans* was purified and characterized in term of its catalytic properties and stability. It displayed highest catalytic efficiency at 60 °C after 10.0 min within acidic pH environment (pH 5). The β-galactosidase exhibited 100% and 60% catalytic activity at 40 °C and 50 °C, respectively even after 120.0 min. The β-galactosidase activity was remained stable in the presence of Zn^2+^, Ni^2+^, and Mg^2+^ ions. The activity was also retained in all investigated organic solvents except DMSO at various ionic concentrations. The surfactants Triton X-100 and SDS caused positive impact on the catalytic activity of enzyme at 1.0 mM concentration (Kamran et al. 2019).

Bernal et al (2012) identified a β-galactosidase produced by *Bacillus circulans* which lost about 90% of its activity after 8 h at 55 °C (Bernal et al. 2012). Raol et al (2015) identified a β-galactosidase produced by an *Aspergillus* species that maintained 50% of the residual activity for 30 min at 70 °C, at which temperature the enzyme lost all the catalytic activity in about 5 h. However, for thermostability studies at 50 °C relative enzymatic activity decreases to about 40% after 3 h (Raol et al. 2015).

Regarding the industrial applicability of this enzyme, it is likely that β-gal is an enzyme as efficient as others already reported in the literature. Shaikh et al. (1999) published for *Rhizomucor* β-galactosidase K_m_ of 1.32 mM toward the ONPG substrate and K_m_ of 50 mM for lactose (Shaikh et al. 1999). O’Connell and Walsh (2008) isolated two β-galactosidases from *A. carbonarius* where the K_m_ values for ONPG were 2.23 and 0.56 mM, and for lactose 82.68 and 308.9 mM. The V_max_ values for the same enzymes were 1.20 and 75 UI/mL toward ONPG and 146 UI/mL and 9.3 UI/mL for lactose (O’Connell and Walsh 2008). O’Connell and Walsh (2010) reported K_m_ of 1.74 mM and V_max_ of 137 UI/mL for ONPG, and for lactose, K_m_ of 48.07 mM and V_max_ of 16 UI/mL in a study on *A. niger* β-galactosidase (O’Connell and Walsh 2010). It is important to have an enzyme acidic resistant for ~ 90 min (the gastric environment of the enzyme remains buffered at acidic pH for this period), so that it reaches the intestine (lactose hydrolysis site) with its preserved catalytic activity. Most commercial preparations do not fully meet the criteria for an optimal supplement. They require enteric coating to protect the supplemental enzyme from the effects of low gastric pH and necessitate higher doses to ensure the required hydrolysis. O’Connell and Walsh (2006) tested in vitro whether the products marketed as an enzymatic supplement of β-galactosidase are compatible with the digestive conditions; they concluded that most of the enzyme preparations were sensitive to gastric acidic pH, thereby retaining only 65% of the enzymatic activity after exposure to digestive-tract conditions. Still, the researchers cautioned that to ensure the degree of hydrolysis required for a dairy-based meal, the number of capsules ingested would have to be increased over the amount recommended by the manufacturer. In the study by O’Connell and Walsh (2008), when β-galactosidase of the *A. carbonarius* was subjected to gastric conditions, it retained 70% of its activity after 2 h of incubation (O’Connell and Walsh 2008).

Thus, achieving enzymatic stability of β-galactosidase in vitro against the hostile gastric conditions is relevant both to guarantee optimal hydrolysis of lactose in vivo and to achieve commercially viable β-galactosidase preparations (coating free).

In addition, the enzyme has physicochemical characteristics and thermal stability and yields gastric simulation test results favorable for its applicability in the pharmaceutical and food industries. Further studies are needed to complement the results obtained in this work. Nevertheless, this present study introduces a filamentous fungus strain from Brazilian Savannah as a good candidate for the production of β-galactosidase which t may have properties of industrial value.

The fungal β-galactosidase in this work was obtained with few purification steps from a low-cost residue, thus being valuable for the industry from the economic point of view. Therefore, the continuation of this enzyme characterization is relevant for industrial application.
